# Myocardial T1 mapping at 3.0T using inversion recovery FLASH readout

**DOI:** 10.1186/1532-429X-17-S1-W2

**Published:** 2015-02-03

**Authors:** Jiaxin Shao, Kim-Lien Nguyen, Peng Hu

**Affiliations:** 1 Department of Radiological Sciences, David Geffen School of Medicine, University of California, Los Angeles, CA, USA; 2 Department of Medicine, Division of Cardiology, David Geffen School of Medicine, University of California, Los Angeles, CA, USA; 3 Biomedical Physics Inter-Departmental Graduate Program, University of California, Los Angeles, CA, USA

## Background

Myocardial T1 mapping methods such as modified Look-Locker inversion-recovery (MOLLI) typically use balanced steady state free precession (bSSFP) readout, which is known to be particularly sensitive to off resonance and thus limits its utility at higher field strengths. Fast low angle shot (FLASH) imaging is known to be more robust against susceptibility artifacts than bSSFP. However, the T1 mapping algorithms that have been developed for bSSFP readout are not directly applicable to FLASH-based T1 mapping sequences at 3.0T. We sought to develop a FLASH-based MOLLI T1 mapping technique for 3.0T and validate it on a cohort of healthy volunteers.

## Methods

The FLASH-MOLLI sequence was developed by modifying the standard MOLLI sequence to use FLASH readout, incorporating a modified T1 estimation algorithm. The proposed algorithm calculates the one dimensional longitudinal signal evolution of the FLASH-MOLLI sequence based on Bloch equations, considering the incomplete inversion using inversion factor δ and the saturation effects of each RF excitation using apparent flip angle α. The 3-parameters (M0, T1, and α, assuming δ is known) or 4-parameters fitting can be performed by using the Levenberg-Marquardt algorithm such that the calculated signal matches best with the measured signal for each pixel.

The FLASH-MOLLI was evaluated against the standard bSSFP-MOLLI based on studies over six phantoms and 10 healthy volunteers in a 3.0T MR scanner, using the same 5-(3)-3 acquisition scheme. For T1 estimation, the proposed 3-parameters fitting was applied to FLASH-MOLLI, and the standard MOLLI fitting with inversion factor correction was applied to bSSFP-MOLLI. Reference T1 values of phantoms were determined by spin-echo experiments. The average inversion factors for phantoms and *in vivo* were determined by "FLASH-MOLLI+M0" with 4-parameters-fitting, which acquires additional M0 weighted image 5 seconds following the 5-(3)-3 acquisition. Based on results measured by the "FLASH-MOLLI+M0" sequence, the inversion factor was set to be 0.955 for phantom studies and 0.88 for in vivo studies for both the FLASH-MOLLI and bSSFP-MOLLI.

## Results

**Figure 1:**
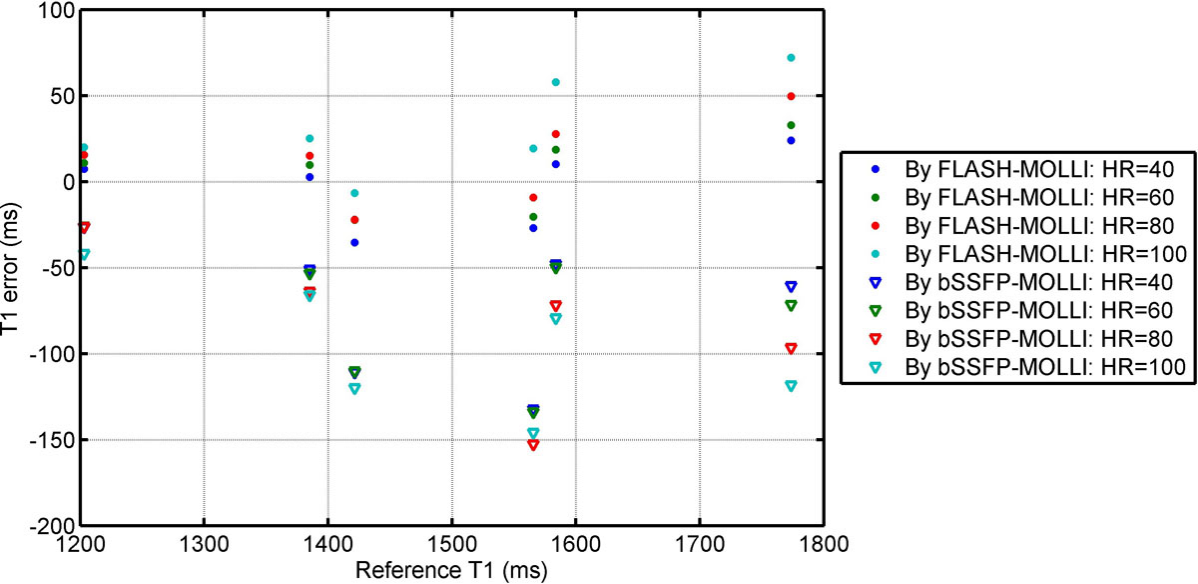
The T1 estimation error at different heart-rates by the FLASH-MOLLI and the standard bSSFP-MOLLI in phantom studies. Inversion factor of 0.955 was used in the FLASH-MOLLI T1 estimation and in the standard MOLLI for inversion factor correction. Compared to the standard MOLLI, FLASH-MOLLI yielded lower T1 estimation errors.

**Figure 2:**
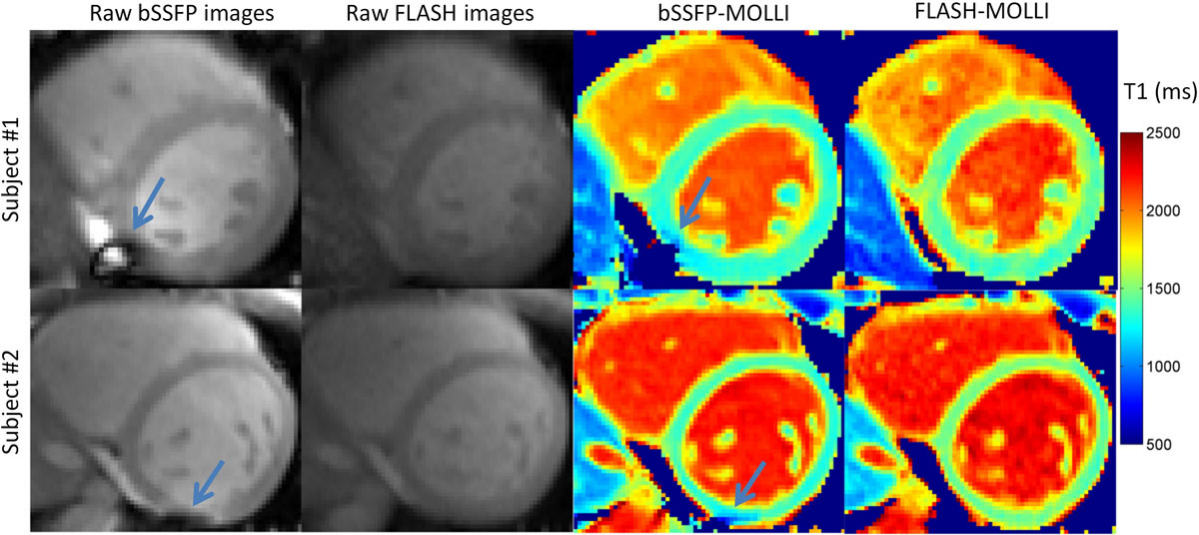
Example of raw bSSFP-MOLLI and FLASH-MOLLI images and T1 maps of the mid left ventricular short axis at 3.0T in a 27 year old male healthy volunteer (subject 1) and a 31 year old female healthy volunteer (subject 2). Acquired raw bSSFP-MOLLI images (first column) showed severe off-resonance artifacts in the inferior/ inferior lateral wall, while acquired raw FLASH-MOLLI images (second column) avoided the artefacts. Corresponding bSSFP-MOLLI T1 maps (third column) showed reduced and inhomogeneous T1 values in the same region (arrow indicated region). FLASH-MOLLI enabled more accurate and homogeneous T1 estimation by rendering off-resonance artifact obsolete.

In phantom studies, even after inversion factor correction, the standard bSSFP-MOLLI produced -82.4±39.8 ms T1 estimation error on average for T1 of 1203-1774 ms and heart rates (HRs) of 40-100 bpm. The FLASH-MOLLI approach reduced the average error to 11.5±26.7 ms (Fig.1). Based on data from 10 volunteers, the native myocardial T1 values by the FLASH-MOLLI were significantly greater than that by the standard bSSFP-MOLLI by 93.1±31.9 ms (1463.8±23.8 ms vs. 1370.7±22.8ms, p<0.001) at 3.0T. Compared to the standard bSSFP-MOLLI sequence, the FLASH-MOLLI sequence is less sensitive to off-resonance artifacts, and provide more accurate and homogeneous in vivo T1 estimations at 3.0T (Fig.2).

## Conclusions

The FLASH-MOLLI approach yields more accurate T1 estimation than the standard bSSFP-MOLLI, and eliminates the banding artifacts associated with bSSFP at 3.0T.

## Funding

N/A.

